# Scanning electron microscope evidence of telocytes in vasculature

**DOI:** 10.1111/jcmm.12333

**Published:** 2014-06-10

**Authors:** Hua Li, Shanshan Lu, Hongnian Liu, Junbo Ge, Hongqi Zhang

**Affiliations:** a Shanghai Institute of Cardiovascular Diseases, Zhongshan Hospital, Fudan UniversityShanghai, China; b Department of Physiology and Medicine/Cardiology, University of CaliforniaLos Angeles, CA, USA; c Department of Anatomy, Histology and Embryology, Shanghai Medical College of Fudan UniversityShanghai, China; d Department of Urology, Affiliated Hospital of Taishan Medical UniversityTaian, China; e Institutes of Biomedical Sciences, Fudan UniversityShanghai, China; f Key Laboratory of Medical Imaging Computing and Computer Assisted Intervention of ShanghaiShanghai, China

**Keywords:** telocytes, telopodes, scanning electron microscopy, arteries, endothelium

## Abstract

Here, we here present scanning electron microscope data for the existent telocytes (TCs) on the endothelial surface of the wall of pig coronary arteries, internal thoracic arteries and carotid arteries. These cells have a small (8.39 ± 1.97 μm/4.95 ± 0.91 μm) cell body of different shapes (from round to triangular, depending on the number of cellular prolongations) with very long (of about 30 μm) and thin cellular processes called telopodes (Tps), which have uneven calibre. The number of Tps ranges between 2 and 6. Tps typically present the alternation of podoms and podomers, and also have a dichotomic branching pattern. These data could influence the current attempts for elucidating the role(s) of TCs.

Telocyte (TC) is a novel type of interstitial cell recently described [1] in stromal connective tissue of many organs [2–13]. The key features of these cells are their small cell body with very long prolongations of uneven calibre, termed telopodes (Tps). In fact, Tps are constituted by an alternation of dilated segments (podoms – which are harbouring mitochondria, endoplasmic reticulum and caveolae) and thin segments (podomers) [1]. The special relation between TCs and blood vessels was documented in different organs [1,7]. Moreover, the pro-angiogenetic behaviour of TCs and their close spatial relationships with newly formed blood vessels was outlined within the border zone of myocardial infarction [14]. By scanning electron microscope (SEM), this study aimed to show visual evidence for the presence of TCs within the vasculature.

The study was approved by the Institutional Ethics Board of Fudan University, according to the generally accepted international standards. Anaesthetized four pigs were fixed and the thoracic cavity was opened, through a puncture in the left ventricle; 3500 ml of heparin physiological saline and 1000 ml 4% paraformaldehyde solution were perfused at physiological pressure respectively. After the perfusion, different arterial segments of large and middle-sized arteries were removed and prepared for scanning electron microscopy according to routine treatment. Endothelial surface of various arteries was examined and the images were captured by using Philips XL30E SEM.

Under SEM, Figure 1 shows numerous TCs on the endothelial surface of medium-sized arteries (internal carotid artery, coronary artery, internal thoracic artery). Even at relatively lower magnification (Fig. 1A), the presence and spatial distribution of very long and thin cellular prolongations (Tps) are obvious, and also their uneven calibre. The alternation of thin segments (podomers) and thick segments (podoms) became more obvious under a higher magnification of an area (white rectangle) of the endothelial surface (Fig. 1B). The measured length of the visible Tp is 11.9 μm. TC cell bodies and Tps seem to be adherent to the endothelial surface. Occasionally, some Tps might detach from the endothelium and freely float upwards in the vascular lumen, in a ‘crab-like’ pattern (Fig. 2C). The density of TCs in blood vessels is different, by region. TCs appear either singular or in groups (Figs 1–4). Irrespective of their location, TCs present various cell body shapes (from round/fusiform to polygonal) depending on the number of Tps they have (Figs 1–4). In the studied tissue samples, the number of Tps varied from one to six (Figs 2–4). The silhouette and the length of Tps appear variously, being irregularly straight and/or curved, the longest measured Tp being of ∼30.0 μm (Fig. 4B). Dichotomic pattern of bifurcation of TPs was observed in Figures 3B and 4C). On the other hand, short and very thin cell processes were observed on the surface of cell bodies of TCs (Figs 2D and 3B). Moreover, contacts between Tps of two distinct adjacent TCs are observed in Figure 2B. Forty TCs were randomly chosen, and the length and width of cell bodies were measured under the SEM screen by the self-carried software of Philips XL30E SEM. The average measured length and width of cell bodies were 8.39 ± 1.97 μm and 4.95 ± 0.91 μm respectively.

**Fig. 1:**
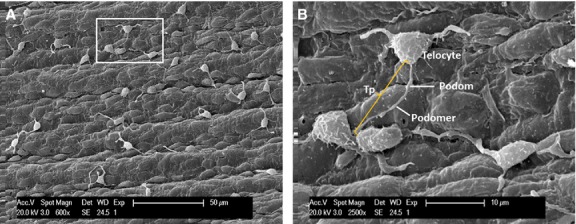
Distribution of vascular telocytes (TC) in pig, scanning electron microscope. (**A**) Under lower power, several TC are observed on the endothelial surface. (**B**) Local enlargement of white rectangle of **A** indicates a typical TC with triangular cell body and one long Telopode (Tps) (Tp, 11.9 μm in length) with alternation of podomers and podoms.

**Fig. 2:**
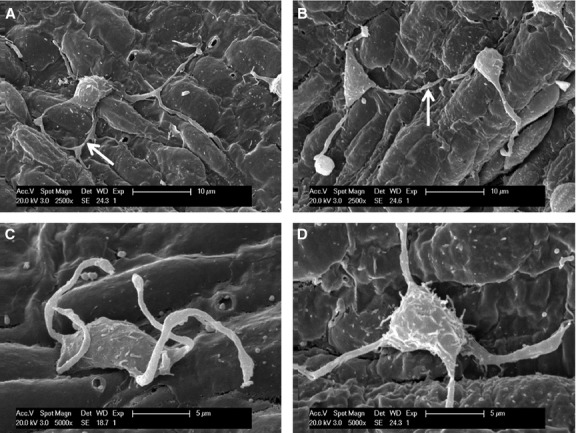
The different types of Telopode (Tps) contacts and short prolongations, scanning electron microscope images of medium-sized arteries in pig. (**A**) Telocyte (TC) has four Tps, and two of them form direct contact and exist in the shape of ring (arrow). (**B**) Two Tps from different TCs form point contacts (arrow). (**C**) The Tps detach from the endothelial surface. (**D**) The shorter and thinner prolongations of cell body of a TC. They are apparently different from Tps and microvilli.

**Fig. 3:**
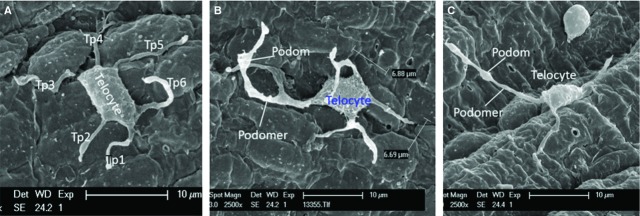
Scanning electron microscope images of number and feature of Telopodes (Tp) adhering to endothelium of medium-sized arteries in pig. (**A**) The Telocyte (TC) with six Tps (Tp1–Tp6). (**B**) The TC with four Tps, three of them form bifurcations and present alternating podomers and podoms. The TC body is 6.88 μm and 6.69 μm in length and width respectively. (**C**) The TC with three Tps, the distinctive Tps are observed with typical podomer and podom.

**Fig. 4:**
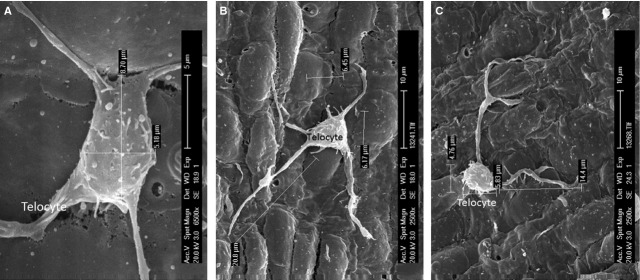
The size of Telocyte (TC) and its Telopodes (Tps). Scanning electron microscope images of medium-sized arteries in pig. (**A**) The TC body looks rectangular and the long and short diameters of the cell body are 8.70 and 5.18 μm respectively. (**B**) The TC with five Tps. The longest Tp is 20.8 μm. (**C**) Two Tps give rise to bifurcations. The cell body is 5.83 μm and 4.76 μm in length and width respectively. The shorter Tps is 14.4 μm in length.

Recently, TCs were found in various organs and tissues, such as skin [2], brain [5], eye [6], skeletal muscle [5], respiratory tract [6], heart [7,8], digestive system [9] and accessory glands of the digestive system [10], genital tract [11,12] and urinary tract [13]. Currently, the establishing of the biological features and the functions of TCs represents a ‘challenge’. In current study, we used SEM to provide visual evidence for the presence of TCs in the vasculature. We studied both large arteries (different divisions of aorta) and middle-sized arteries (internal thoracic arteries, common carotid arteries and coronary arteries, *etc*.). We showed the presence of a new population of cells (TCs) on the pig endothelial surface with the same ultrastructural features as TCs, previously described by Popescu's group [1]. We found TCs only in the medium-sized arteries, rather than in large arteries. This might be because of the higher shear stress that occurred in these vascular regions, and could be speculated as TCs are less tolerant for turbulent blood flow. On the other hand, it is well known that living cells, for self-assuring their survival, have their natural tendency for minimizing their surface in the liquid environment. Thus, TCs in vasculature could appear with slightly modified morphology, with more spherical, shorter and thicker prolongations. In summary, we present visual evidence for the existence of TCs in the vasculature. The morphological specificity of vascular TCs might be associated with the dynamic environment where they exist. Further morphological and functional correlations need to be established.
